# A Clinical Breathomics Dataset

**DOI:** 10.1038/s41597-024-03052-2

**Published:** 2024-02-14

**Authors:** Ping-Hung Kuo, Yue-Chen Jhong, Tien-Chueh Kuo, Yu-Ting Hsu, Ching-Hua Kuo, Yufeng Jane Tseng

**Affiliations:** 1 National Taiwan University Hospital, No. 1, Changde St., Zhongzheng Dist., Taipei City, 100229 Taiwan; 2 Graduate Institute of Biomedical Electronics and Bioinformatics, College of Electrical Engineering and Computer Science, National Taiwan University, No. 1, Sec. 4, Roosevelt Road, Taipei, 10617 Taiwan; 3 The Metabolomics Core Laboratory, Center of Genomic Medicine, National Taiwan University, No. 1, Sec. 4, Roosevelt Road, Taipei, 10617 Taiwan; 4 Drug Research Center, College of Pharmacy, College of Medicine, National Taiwan University, No. 33, Linsen S. Road, Taipei, 10055 Taiwan; 5 Department of Pharmacy, School of Pharmacy, College of Medicine, National Taiwan University, No. 33, Linsen S. Road, Taipei, 10055 Taiwan; 6 Department of Computer Science and Information Engineering, College of Electrical Engineering and Computer Science, National Taiwan University, No. 1, Sec. 4, Roosevelt Road, Taipei, 10617 Taiwan

**Keywords:** Biomarkers, Metabolomics

## Abstract

This study entailed a comprehensive GC‒MS analysis conducted on 121 patient samples to generate a clinical breathomics dataset. Breath molecules, indicative of diverse conditions such as psychological and pathological states and the microbiome, were of particular interest due to their non-invasive nature. The highlighted noninvasive approach for detecting these breath molecules significantly enhances diagnostic and monitoring capacities. This dataset cataloged volatile organic compounds (VOCs) from the breath of individuals with asthma, bronchiectasis, and chronic obstructive pulmonary disease. Uniform and consistent sample collection protocols were strictly adhered to during the accumulation of this extensive dataset, ensuring its reliability. It encapsulates extensive human clinical breath molecule data pertinent to three specific diseases. This consequential clinical breathomics dataset is a crucial resource for researchers and clinicians in identifying and exploring important compounds within the patient’s breath, thereby augmenting future diagnostic and therapeutic initiatives.

## Background & Summary

Breathomics is a field of research that examines the metabolic activity in a system through the analysis of volatile organic compounds (VOCs)^1^. VOCs are highly volatile, gaseous organic molecules that can reflect the metabolic activity in the human body or the interaction between the human body and the environment through inhaled air, food, drink, and drugs^2^. In respiratory diseases, the close contact between VOCs and the respiratory tract makes them an important compound for understanding airway diseases^1–7^ or lung cancer^2,8,9^.

VOCs can be obtained from human exhaled gas or exhaled breath condensate (EBC) samples^10–12^; both of these sampling methods are noninvasive compared to other diagnostic procedures, such as bronchoscopy, bronchoalveolar lavage, and biopsy^2,13^. Exhaled breath is more actively studied than biological samples, such as saliva, breast milk, sweat, epithelial tissue, urine, or feces^14–16^.

Gas chromatography-mass spectrometry (GC‒MS)^14–20^ and electronic noses (eNoses)^21–23^ are two common methods used to analyze VOCs. GC‒MS has high sensitivity and potential for identifying and quantifying unknown components. Nevertheless, its clinical implementation can be complex due to the need for highly trained personnel and the laborious analysis procedure^2,24^. On the other hand, eNoses^2^ are easy to use, cost-effective, and capable of real-time monitoring, but their lack of selectivity and susceptibility to interference can affect their reliability and robustness^14–16^.

In the COVID-19 pandemic, advancements within the realm of breathomics research have been swift and substantial. Despite such progress, a discernible lack of comprehensive datasets dedicated to breath research remains. Recognizing this deficit, we present the clinical breathomics dataset to bridge this gap. The release of this indispensable dataset marks a seminal phase in community sharing for this research domain. It is a valuable asset for further explorations into breath studies, aiding researchers in unraveling the intricate biomedical underpinnings of various diseases. Moreover, this robust dataset is a credible validation tool for ongoing and future breath studies focused on asthma, bronchiectasis, and COPD, further bolstering the field’s collective research endeavors.

## Methods

### Ethics statement

All methods employed in this study complied with relevant guidelines and regulations. The use of the Asthma Control Test (ACT) and Global Initiative for Asthma (GINA) control status was approved by the Research Ethics Committee C of the National Taiwan University Hospital. Participants were recruited from May 2011 to April 2014 and provided written informed consent. The study was registered with ClinicalTrials.gov, with the identifiers NCT01439737 and NCT01410422.

### Study subjects

Subjects of studies can be divided into asthma, bronchiectasis, and COPD. After analyzing breath samples by headspace solid-phase microextraction combined with gas chromatography time-of-flight mass spectrometry (HS-SPME GC-TOF-MS). These clinical data were then combined with previously collected clinical asthma data with the same method described in the following sections. Overall, we have identified 104 VOCs in data from 53 clinical asthma samples, 35 bronchiectasis samples, and 33 COPD samples in this dataset.

### Collection of exhaled breath condensate samples

Samples of exhaled breath condensate (EBC) were collected from healthy individuals using the commercial device RTube^®^ (Respiratory Research, Charlottesville, VA, USA). The subjects were instructed to fast for 8 hours before sample collection. The aluminum sleeve of the device was precooled at −80 °C for 20 minutes before each sample collection. Participants were asked to inhale and exhale through their mouth and breathe tidally for 15 minutes without wearing a nose clip and to temporarily discontinue the EBC collection if they needed to swallow saliva or felt the urge to cough. The exhaled breath was condensed and collected in a polypropylene-based tube, and the EBC samples were stored at −80 °C immediately until analysis. EBC samples collected from 5–7 individuals were pooled as quality control (QC) samples and separated into multiple vials for analytical method development to ensure optimal sample quality. Throughout the entire experiment, we employed the pooled QC samples across batches to uphold a consistent level of quality.

### HS-SPME sampling procedure

Detecting compounds in exhaled breath condensate (EBC) can be challenging due to the samples’ low concentrations of volatile and nonvolatile compounds. Sample preconcentration techniques, such as solid-phase microextraction (SPME), are necessary to overcome this problem. SPME, invented in the late 1980s^25^, offers efficiency, simplicity, and minimal solvent consumption, making it a popular choice for preconcentrating compounds in biological gas matrices^25–30^. The preconcentration mechanism relies on establishing equilibrium between the matrix and a fused silica fiber coating. The analytes are then desorbed from the fiber and injected into gas chromatography (GC) or liquid chromatography.

Before analysis, the EBC sample vials were cleaned twice in a sonicator with deionized water, ethanol, and acetone. The vials were then dried under a nitrogen stream and combined with 0.5 mL of EBC sample and 200 mg of NaCl. The headspace was then sampled using a PDMS/DVB fiber and extracted at 45 °C for 4 hours.

After extraction, the SPME fiber was immediately transferred to the GC injector port at 250°C and heated for 3 minutes in splitless mode to thermally desorb the analytes into the GC column, avoiding the loss of the extracted substances and minimizing analyte evaporation. Before each sample extraction, the SPME fiber was cleaned in the GC injection port at 250°C for 30 minutes to prevent sample carryover. To ensure accuracy, we conducted a blank run to make sure the cleaning process of the fiber was executed thoroughly before analysis.

### GC-TOF-MS analyses

All analyses were performed on a LECO Pegasus 4D time-of-flight mass spectrometer (GC-TOF-MS) (Leco Corporation, St. Joseph, MI, USA). The Pegasus 4D GC-TOF-MS was equipped with Agilent 7890a gas chromatography. The chromatographic column was a 30 m DB-5MS capillary column (5% phenyl, 95% dimethylpolysiloxane) with an internal diameter of 250 μm (Agilent Technologies, Santa Clara, CA). The oven began at a holding temperature of 50 °C for 2 minutes, then increased to 280 °C at 10 °C/min. The temperature was held at 280 °C for 5 minutes. The helium carrier gas flow rate was set at 1 mL/min. The electron energy was 70 eV, and the ion source temperature was 240 °C. The TOF-MS detector was operated at 1500 V and in auto-detection mode. The analytes were acquired in full scan mode with a mass range of 40–550 m/z.

### GC‒MS data analysis and compound identification

Data obtained from the MS analysis, stored in RAW file format, were subjected to processing employing LECO ChromaTOF^®^ software (version 4.33). This software version is specially optimized for enhanced compatibility and functionality with the Pegasus instrument. The cdf files, obtained from different disease groups, were analyzed separately using the eRah R package^31^. This package automates the processes of compound deconvolution, sample alignment, and metabolite identification through GC spectral library matching. The software’s user manual outlines the procedures involved in the analysis, such as deconvolution, alignment, missing compound recovery, and naming. The NIST 20 MS/MS spectral libraries were utilized as the reference GC‒MS library during the identification process for matching spectra.

## Data Records

The clinical breathomics dataset is available as open access on the figshare online repository^32^. This dataset consists of an in-house R script file, a Python script file, a spreadsheet file for metadata, three comma separate values (CSV) files and a spreadsheet file for the intersection of the detected compounds (gcms_analysis.R, heatmap.py, CBD_metadata_for_ver3.xlsx, Asthma_peaktable_ver3.csv, Bronchi_peaktable_ver3.csv, COPD_peaktable_ver3.csv and Intersection_of_detected_compounds.xlsx)

**gcms_analysis.R** - an R script for GC-MS data analysis.

**heatmap.py** – a Python script for performing the heat map analysis from 3 peak tables.

**CBD_metadata_for_ver3.xlsx** – a spreadsheet file for metadata including gender, age, ACT (Asthma Control Test) score, CAT (COPD Assessment Test) score, and the pulmonary function data.

**Asthma_peaktable_ver3.csv** – a peak table with 131 rows (metabolites) and 53 columns (samples). The column headers are patients’ IDs. The first column is the PubChem CID (PubChem Compound Identification), and the second column is the IUPAC name of the chemical compound.

**Bronchi_peaktable_ver3.csv** – a peak table with 120 rows (metabolites) and 35 columns (samples). The column headers are patients’ IDs. The first column is the PubChem CID, and the second column is the IUPAC name of the chemical compound.

**COPD_peaktable_ver3.csv** – a peak table with 123 rows (metabolites) and 33 columns (samples). The column headers are patients’ IDs. The first column is the PubChem CID, and the second column is the IUPAC name of the chemical compound.

**Fig. 1:**
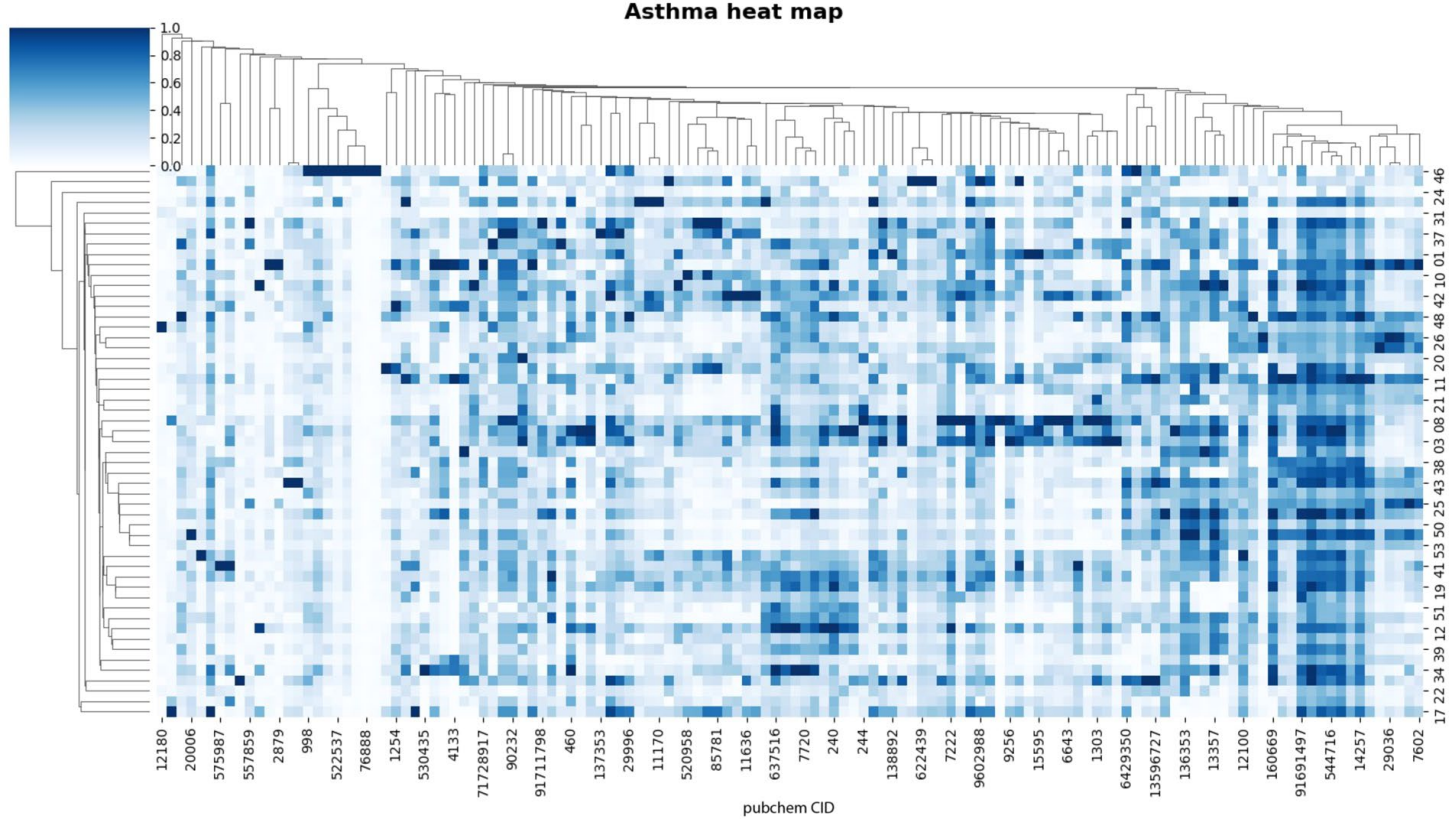
Heat map analysis of 131 metabolites in 53 asthma patients. Each column represents a metabolite, and each row represents a sample. Both rows and columns are clustered using correlation distance and single linkage.

**Fig. 2:**
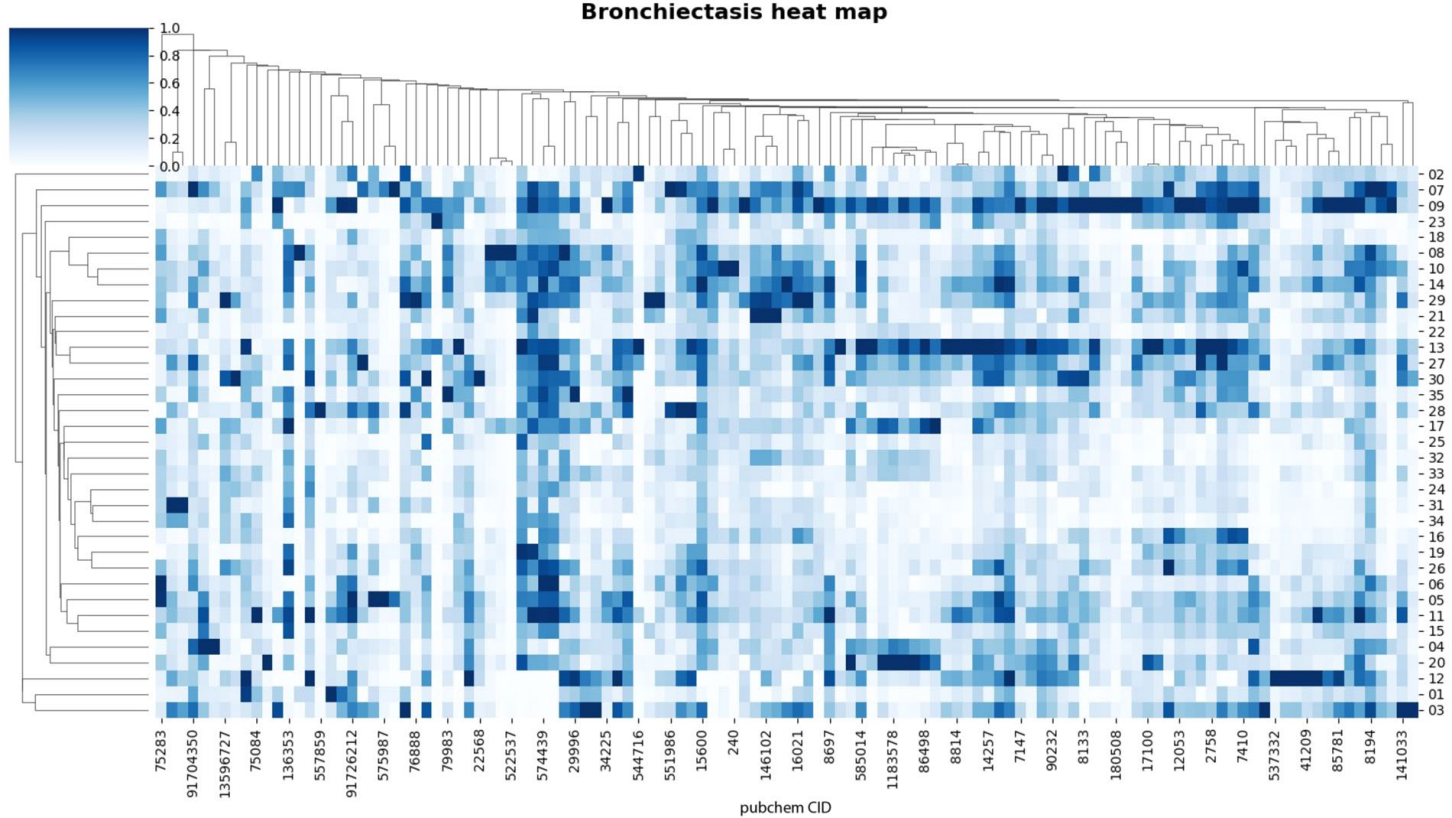
Heat map analysis of 120 metabolites in 35 bronchiectasis patients. Each column represents a metabolite, and each row represents a sample. Both rows and columns are clustered using correlation distance and single linkage.

**Fig. 3:**
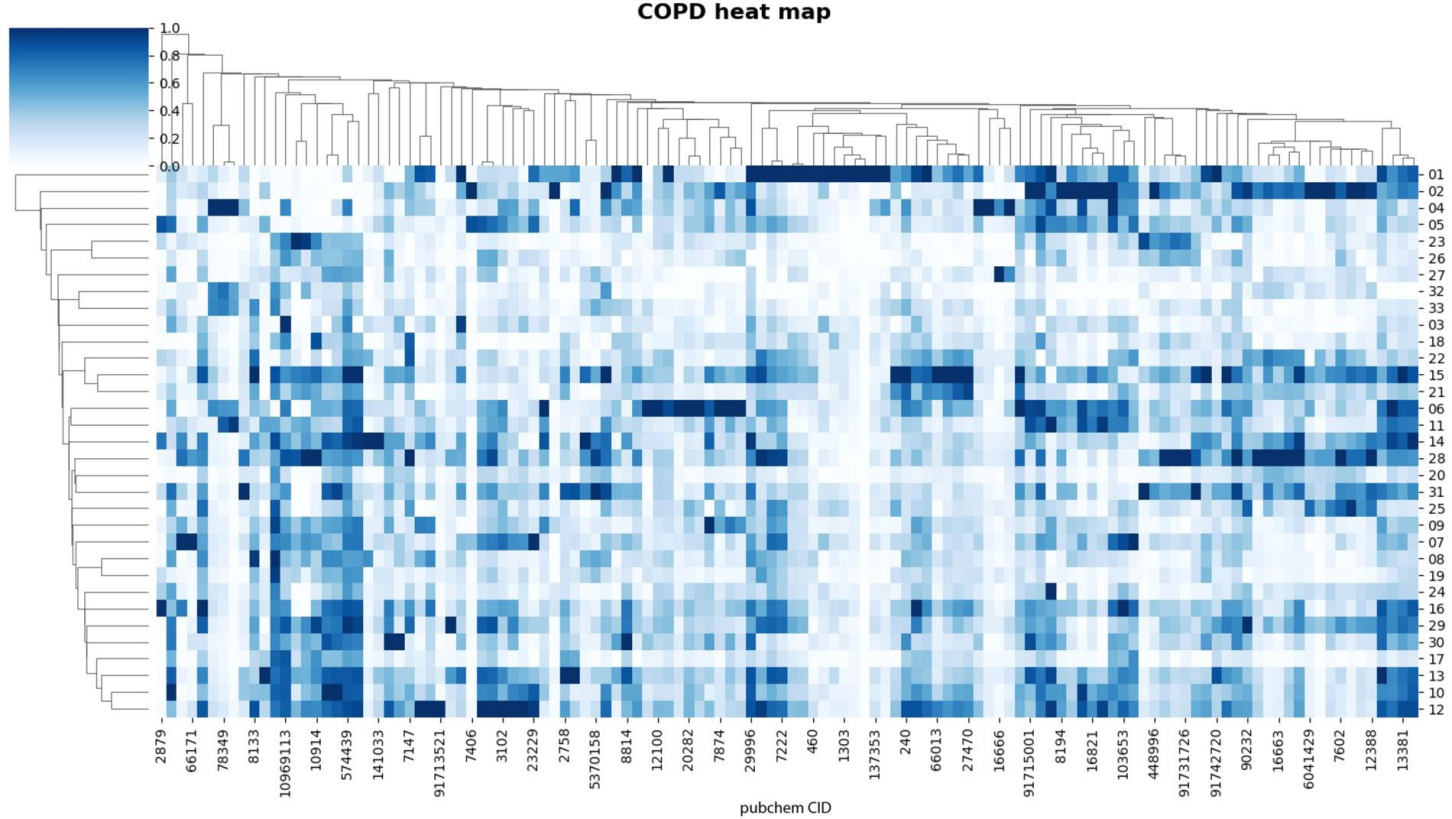
Heat map analysis of 123 metabolites in 33 COPD patients. Each column represents a metabolite, and each row represents a sample. Both rows and columns are clustered using correlation distance and single linkage.

The identified peak tables corresponding to asthma, bronchiectasis, and COPD were represented through heat map visualizations, as depicted in Figs. 1–3.

**Intersection_of_detected_compounds.xlsx** – a spreadsheet file with the intersection of the detected compounds of three peak tables.

### Dataset extraction from clinical gc‒ms data analysis

The peak table for each disease group was compiled manually by merging the results from the ‘alignList’ and ‘idList’ generated by the eRah analysis (generated from the alignment and identification processes, respectively). The peak table includes information about the most closely matched compound name, the PubChem CID, the clinical sample group, and the chromatographic peak intensity of each identified compound obtained after deconvolution. To better visualize the relationship between compounds and diseases, each clinical sample’s chromatographic peak intensities were scaled using min-max scaling (ranging from 0 to 1). The scaled peak table was then used to generate group box plots and dot plots using the R package ‘ggplot’ to depict the scaled intensity of each identified compound for the three disease groups (asthma, bronchiectasis, and COPD). 131, 120, and 123 compounds were identified in the asthma, bronchiectasis, and COPD groups, respectively. The intersection of the compounds of three peak tables is displayed in a spreadsheet file in the figshare repository.

## Technical Validation

The compounds identified from our clinical GC‒MS analysis were detected and consistent with some published literature sources. Our results were consistent with the presence of undecane^22^,^33^, 1-ethyl-3-methyl benzene^34^, and cyclohexane^35^ as important compounds for COPD in previous studies. They showed that n-heptane could distinguish between VOC patterns in patients with acute exacerbation of COPD (AECOPD) and stable COPD^35^. Additionally, decane was shown to be associated with oxidative stress and inflammation^36^, making it an important compound for asthma screening.

## Usage Notes

The clinical breathomics dataset consists of 3 peak tables of the EBC samples from asthma, bronchiectasis, and COPD subjects and a spreadsheet file for metadata. Furthermore, it is important to acknowledge that the pulmonary function data contained in the metadata could impact the volume of exhaled breath and subsequently influence the detected intensity of the VOCs. Therefore, we suggest that the pulmonary function data should be taken into account and the total data scaling and normalization should be conducted in the pre-processing. For the missing value in the peak tables, we recommend doing missing value imputation before statistical analyses.

## Data Availability

The in-house R and Python scripts for GC-MS and heat map analysis are available in the figshare repository (10.6084/m9.figshare.23522490.v6).
